# Comparative evaluation of the CGuard dual−layer stent and Carotid Wallstent for elective carotid artery stenting: a retrospective multicenter study

**DOI:** 10.1007/s00234-025-03764-1

**Published:** 2025-09-08

**Authors:** Yves Leonard Voss, Mousa Zidan, Alexander Radbruch, Igor Fischer, Franziska Dorn, Hannes Nordmeyer

**Affiliations:** 1 Department of Diagnostic and Interventional Neuroradiology, University Hospital Bonn, Bonn, Germany; 2 Department of Diagnostic and Interventional Neuroradiology, Klinikum Solingen, Solingen, Germany; 3 Neurovascular Research Unit, Heinrich Heine University Düsseldorf, Düsseldorf, Germany; 4 School of Medicine, Department of Health, Witten/Herdecke University, Witten, Germany

**Keywords:** Carotid artery revascularization, Carotid artery stenting, Dual-layer mesh stent

## Abstract

**Purpose:**

This study aims to evaluate the safety and efficacy of the CGuard dual-layer stent with its mesh embolic protection system (EPS) in elective cases for treatment of internal carotid artery stenosis and compares it to the Carotid Wallstent as benchmark.

**Methods:**

In this retrospective, multicenter study, we analyzed data from consecutive patients who underwent carotid artery stenting with CGuard at two high-volume neurointerventional centers and compared them with prior consecutive patients treated with Carotid Wallstent (CWS), with and without a balloon guiding catheter (BGC) as protection, at the same institutions. Patient demographics, procedural details, clinical complications, early in-stent thrombosis and occlusion rates, and late follow-up restenosis rates were assessed.

**Results:**

A total of 428 patients were treated, 144 with the CGuard stent, 203 with CWS + BGC and 83 with CWS-BGC, the majority of patients for symptomatic stenoses. Technical success was achieved in 98.6% of CGuard patients. No clinical complications were observed in CGuard patients, however the clinical complication rate was 2.96% (6/203) for CWS + BGC and 4.94% (4/83) for CWS-BGC patients (*p* = 0.052). The in-hospital stent occlusion rate was 0.69% (1/144) for CGuard and 2.1% (6/286) in CWS ± BGC patients (*p* = 0.49). On long-term follow-up (mean 9.9 months) the CGuard demonstrated a comparatively low rate of restenosis (6.25%) and retreatment (2.1%).

**Conclusion:**

The CGuard dual-layer stent was safe and effective for carotid artery stenting in our series. Its design appears to contribute to a low risk of periprocedural complications, high technical success rate, while maintaining restenosis rates comparable to the Carotid Wallstent.

## Introduction

Carotid artery stenoses are the cause of approximately 20% of all ischemic strokes [1, 2]. Carotid artery stenting (CAS) is an endovascular alternative to carotid endarterectomy (CEA) for treating stenoses of the Internal carotid artery (ICA). While the overall long-term outcomes of CAS and CEA procedures are comparable [3–5] there is an ongoing debate about which patient benefits most from which of the two treatment modalities. The key differences established between the two treatment options today are the risks during and after the procedures: While CAS is associated with a higher rate of periprocedural carotid-embolic ischemic lesions compared with CEA within 30-days after the procedure, the periprocedural rate of myocardial infarctions is lower for patients undergoing CAS [6–8].

Thromboembolic events can occur at several stages of the CAS procedure: During catheterization of the target artery, while the stenosis is being passed with the microwire and stent applicator, during stent release and during pre- and post-dilatation. Furthermore, in-stent thrombus formation due to the thrombogenicity of the stent itself or protruded plaque components, may occur in the postinterventional phase until reendothelialization of the stent is complete. Different embolic protection strategies were developed with the aim to reduce the risk of thromboembolic events associated with CAS [8].

Plaque protrusion through the stent struts is a potential source of emboli in the early period after CAS and the majority of adverse neurological events occur within 30 days after the procedure adding to the overall risk of CAS [9]. De Donato et al. [10] found plaque prolapse in up to 68% of CAS patients who were treated with open cell design stents using optical coherence tomography. Plaque protrusion is not prevented by procedural protection devices such as balloon guide catheters and filter wires.

The CGuard stent is a new generation dual-layer mesh stent (DLMS): It consists of an inner open-cell stent and an outer mesh layer that serves as a built-in embolic protection system to protect from plaque protrusion with subsequent embolization during and after CAS by improving plaque coverage. Both properties are combined to address the disadvantages of classical open-cell-stents allowing plaque protrusion through their large free-cell areas, as well as some closed-cell stents disadvantages of low flexibility adding to technical problems in stent delivery and rigidity after implantation [11]. The CGuard stent can be used with or without additional protection systems such as balloon guide catheters.

The aim of this retrospective multicentric study was to evaluate the efficacy and safety of the CGuard stent in a real-world scenario.

## Methods

### Patient Selection

This study is a retrospective, observational evaluation of patients who underwent CAS between January 1, 2018, and December 31, 2022, at two German high-volume neurointerventional centers. We reviewed patients’ angiographic images and an anonymized database containing information from clinical records and follow-up ultrasound exams.

### Devices

The CGuard EPS stent consists of a self-expandable nitinol open-cell laser cut stent that is externally wrapped with an additional mesh layer woven from a single 20 μm thickness Polyethylene Terephthalate (PET) strand with mesh pore sizes of 150–180 μm in a fully expanded stent according to the manufacturer InspireMD. It is mounted on a self-expandable rapid exchange delivery system with a tapered tip olive and available in diameters of 6 to 10 mm and lengths of 20 to 60 mm.

Sizes used in the study were (nominal diameter/length): 6/30, 6/40, 7/30, 7/40, 8/30, 8/40, 10/30, 10/40, 10/60. A CGuard diameter of 6 mm was used in 9 patients, 7 mm in 20 patients, 8 mm in 77 patients, 10 mm in 38 patients. A CGuard length of 30 mm was used in 23 patients, 40 mm in 110 patients and 60 mm in 13 patients.

The Carotid Wallstent (CWS) is a self-expanding, closed-cell stent braided from cobalt-chromium-iron-nickel-molybdenum alloy wires with a radiopaque tantalum core (drawn filled tubing) with a pore size of 900–1500 μm and mounted on a rapid exchange delivery system and is one of the most used stents for CAS procedures in Europe in the last two decades. It is known for its comparatively high radial strength when implanted but lower flexibility and shortening of its visible length upon deployment [12].

### Endovascular Procedure

Baseline investigation included neurological assessment and carotid ultrasound, cases were planned after vascular board approval. Patients with all aortic arch types were included, patients with concurrent stenoses or occlusions of other supraaortic vessels were not excluded.

All CAS procedures were performed by board-certified neurointerventionalists on biplane angiographic systems (Philips, Netherlands; Siemens healthineers, Germany). Iodinated contrast agents were used for angiography. A biplane angiographic projection of the intracranial circulation was acquired before passing the ICA lesion and at the end of the procedure in all cases. Cerebral embolism on cerebral angiogram was noted if present.

All procedures were carried out via femoral access using introducer sheaths (8 Fr). Based on availability and institutional standards different non-balloon guiding catheters (Fubuki, Guider Softip, Cerebase, Vista Brite tip) were used. As protection system the balloon guiding catheter Flowgate II was used in all + BGC cases, a filter wire was not used, according to institutional standards.

The common carotid artery was catheterized using a 0,035” wire, the ICA stenosis was passed with a 0,014” wire in all cases.

Pre-dilatation was performed only when deemed necessary at the operator’s discretion. Stent delivery was routinely followed by balloon post-dilatation. All CAS procedures were performed according to the devices’ specific instructions for use. General anesthesia was performed according to institutional standards or when necessary due to patient specific circumstances.

### Medical treatment

In all patients, dual antiplatelet therapy was initiated at least 24 h before the procedure. In case of clopidogrel the loading dose was 600 mg. Intraprocedural anticoagulation was achieved using 3.000 IU to 5.000 IU of heparin (UFH). Post-operative medical therapy included Clopidogrel 75 mg per day for one to four months upon the operator’s discretion and lifelong ASA 100 mg per day.

### Patient follow-up

All patients underwent post-procedural neurological evaluation by a board-certified neurologist. Stent patency was systematically assessed by an experienced board-certified stroke neurologist using ultrasound. Patients were then scheduled for ultrasound follow up within 6 to 12 months after treatment. Adverse events were routinely recorded.

Restenosis was defined as a reduction of the target artery diameter of at least 70% and a peak systolic velocity of more than 300 cm/s in ultrasound served to indicate a > 70% stenosis.

## Outcome Evaluation

### Statistical analysis

Quantitative variables were described by means and standard deviation. A T-test was used for real valued independent variables, a Chi-square test was used for nominal variables. A logistic regression analysis was performed for binary outcome and real valued independent variables. A p value of less than 0.05 was considered statistically significant. Statistical analyses were performed in ScyPy (Python, Delaware, USA).

## Results

### Patient characteristics and procedural situation

Baseline Demographic and procedural characteristics of the study population are reported in Table 1.

**Table 1:** Demographic characteristics, procedural data and follow-up results

Variables Mean ± STD or % (*n*/*N*) or median (IQR)	CGuard (*n* = 144)	CWS + BGC (*n* = 203)	CWS-BGC (*n* = 81)	*P* value
Patient Characteristics				
Age, yrs	70.8 ± 8.2	72.4 ± 8.9	70.1 ± 8.3	0.13
Male Gender Female Gender	63.9% (92/144) 36.1% (52/144)	62.1% (126/203) 37.9% (77/203)	64.2% (52/81) 35.8% (29/81)	0.67
General Anesthesia Local Anesthesia	42.4% (61/144) 57.6% (83/144)	79.3% (161/203) 20.7% (42/203)	44.4% (36/81) 55.6% (45/81)	< 0.001
Right Side Left Side	44.4% (64/144) 55.6% (80/144)	47.8% (97/203) 52.2% (106/203)	49.4% (40/81) 50.6% (41/81)	0.74
Stenosis Grade (NASCET)	75.5% ± 14.2	81.7% ± 11.0	80.3% ± 10.0	0.003
Atherosclerotic Stenosis Post-TEA Restenosis	90.3% (130/144) 9.7% (14/144)	97.0% (197/203) 3.0% (6/203)	91.4% (74/81) 8.6% (7/81)	0.024
Symptomatic Status	60.4% (87/144)	79.3% (161/203)	67.9% (55/81)	< 0.001
Stent & procedure				
Stent size Stent length	8 mm (IQR = 7–10) 40 mm (IQR = 40–40)	9 mm (IQR = 7–9) 40 mm (IQR = 40–40)	7 mm (IQR = 7–9) 40 mm (IQR = 30–40)	0.017 0.025
Pre-dilatation Pre-dilatation Balloon size (mm)	11.1% (16/144) 3 mm (IQR = 3–3)	11.3% (23/203) 3 mm (IQR = 3–3.5)	33.3% (27/81) 5 mm (IQR = 3–5)	< 0.001 0.029
Post-dilatation Post-dilatation Balloon size (mm)	92.4% (133/144) 5.5 mm (IQR = 5.0–5.5)	97.5% (198/203) 5.5 mm (IQR = 5.0–5.5)	86.4% (70/81) 5.0 mm (IQR = 5.0–5.5)	0.0017 < 0.001
Medication				
DAPT duration (months)	1 (IQR = 1–3) 1.9 ± 1.1	1 (IQR = 1–1) 1.0 ± 0.0	4 (IQR = 1–4) 2.9 ± 1.3	< 0.001
Triple therapy	2.8% (4/144)	3.0% (6/203)	1.2% (1/81)	0.7
Outcome				
Clinical complication rate	0% (0/144)	2.96% (6/203)	4.94% (4/81)	0.045
Technical difficulties rate	2.08% (3/144)	0.99% (2/203)	4.94% (4/81)	0.11
Intraprocedural In-Stent-thrombosis	0.69% (1/144)	0% (0/203)	0% (0/81)	0.37
Early postop. In-Stent-occlusion (In-hospital)	0.69% (1/144)	0.99% (2/203)	4.94% (4/81)	0.033
Hemorrhage	0.7% (1/144)	1.0% (2/203)	1.2% (1/81)	0.92
In-Stent-Stenosis 24 h	4.2% (6/144)	0% (0/203)	7.4% (6/81)	< 0.001
Late FU available	47.2% (68/144)	46.8% (95/203)	42.0% (34/81)	0.72
Late FU delay (months)	9.9 ± 7.8	13.1 ± 8.1	13.1 ± 9.3	0.51
In-Stent-Stenosis late FU	6.25% (4/64)	9.2% (8/87)	0% (0/34)	0.76
Retreatment until late FU	2.1% (3/144)	3.5% (7/203)	2.5% (2/81)	0.73

*BGC* = Balloon guide catheter. *NASCET* = North American Symptomatic Carotid Endarterectomy Trial. *DAPT* = dual antiplatelet therapy. *FU* = follow-up

The mean age was 70.8 ± 8.2 years in the CGuard group, 72.4 ± 8.9 years in the CWS + BGC and 70.1 ± 8.3 years in the CWS-BGC group. The male gender was predominant in 63.9%, 62.1% and 64.2% of patients respectively. The differences in age were not significant between groups.

General anesthesia was performed in 42.4% of CGuard patients, 79.3% of CWS + BGC patients and 44.4% of CWS-BGC patients – all other patients were treated under local anesthesia. The rate of general anesthesia was significantly higher for the CWS + BGC group than for both other groups (*p* < 0.001).

The mean carotid stenosis grade (NASCET) was 75.5% ± 14.2% for the CGuard group compared to 81.7% ± 11.0% for the CWS + BGC group and 80.3% ± 10.0% for the CWS-BCG group, those differences were significant (*p* = 0.003) overall. Grouped for stent type (CGuard vs. CWS ± BGC) the average degree of stenosis was significantly lower in the CGuard group (*p* = 0.0007), but there was no significant difference (*p* = 0.48) of the stenosis degree in respect to the procedural presence of a protection system (CGuard and CWS + BGC vs. CWS-BGC).

The treated carotid lesion was a restenosis after prior TEA in 9.7% of CGuard patients, in 3% of CWS + BGC patients and in 8.6% of CWS-BGC patients, the remaining lesions were atherosclerotic ICA stenoses. The rate of Restenosis after prior TEA was therefore significantly lower in CWS + BGC patients (*p* = 0.024).

Patients presented with symptomatic stenosis in 60.4% of cases in the CGuard group, 79.3% in the CWS + BGC and 67.9% in the CWS-BGC groups respectively, resulting in a significantly increased frequency of symptomatic status for the CWS + BGC group (*p* < 0.001). Grouped for stent type (CGuard vs. CWS ± BGC) patients were significantly more often symptomatic in the CWS ± BGC than in the CGuard group (*p* = 0.0012), but there was no significant difference (*p* = 0.68) in the rate of symptomatic status in respect to the procedural use of a protection system (CGuard and CWS + BGC vs. CWS-BGC).

## Endovascular treatment details and results

Pre-dilatation was performed in 11.1% of CGuard patients and 11.3% of CWS + BGC patients, but significantly more often in 33.3% of CWS-BGC patients (*p* < 0.001). Post-dilatation was performed in 92.4% of CGuard patients, 97.5% of CWS + BGC patients and significantly less often in 86.4% of CWS-BGC patients (*p* = 0.0017).

Technical difficulties of note without clinical sequelae occurred in 3 patients in the CGuard group (2.08%): Two highly calcified lesions could not be passed with the CGuard device but were then successfully treated with a CWS in one and a Casper stent (Microvention, Irvyne, USA) in the other case. In another case after uneventful implantation of the CGuard the passage of the balloon for post-dilatation was difficult and a dissection occurred, that was then treated with an overlapping CWS. Technical difficulties occurred in two patients in the CWS-BGC group (0.99%) and in four patients in the CWS + BGC group (4.94%): In four cases, a highly calcified lesion could not be passed with the CWS despite pre-dilatation and was then treated with a Casper stent. In one case a hemodynamic post-stent kinking occurred after CWS implantation and was resolved by implantation of an overlapping CGuard. In one case the CWS could not reach its target lesion due to anatomical reasons and the procedure was stopped without CAS. There were no significant differences in the rate of technical difficulties between the three groups overall (*p* = 0.11) or grouped for stent type or the use of a protection system (*p* = 0.12).

Clinical complications during hospital stay were detected in none (0%, 0/144) of the patients in the CGuard group. However, there were six clinical complications in the CWS + BGC group (2.96%, 6/203): One patient passed away due to an acute myocardial infarction three days after the procedure, one patient died from a major intracerebral hemorrhage and one patient experienced ipsilateral stroke with a moderate arm paresis in the post-procedural phase. In three of those six patients the clinical complications were caused by intraprocedural complications: One patient had a distal M3 embolism after passage of the aortic arch detected on cerebral angiogram before balloon or stent utilization, that was treated with mechanical thrombectomy with mild clinical sequelae. One patient suffered from a distal embolism that resulted in mild arm paresis. One patient developed a cerebellar embolic infarction (unrelated to the treated artery territory) attributed to aorto-embolic origin during the procedure. Four patients in the CWS-BGC group experienced clinical complications (4.94%): One myocardial infarction, one hyperperfusion syndrome, one minor and one major ipsilateral ischemic stroke (NIHSS > 3). One of the stroke patients had an intraprocedural complication due to a M2 thromboembolism during the procedure that could be treated with mechanical thrombectomy without clinical sequelae. One patient suffered from a postprocedural embolic infarction. There was no statistically significant difference in the rate of clinical complication between using the CWS ± BGC versus the CGuard stent type (*p* = 0.052). There was no statistically significant difference regarding the use of a protection system (*p* = 0.19).

Intraprocedural In-stent thrombosis occurred in one patient in the CGuard group (0.69%) and completely resolved during the procedure after i.v. Tirofiban was given. This did not result in clinical deterioration. No intraprocedural In-stent thrombosis occurred in the CWS patients. This difference was not significant.

Postprocedural In-Stent occlusion during the hospital stay occurred in one patient in the CGuard group (0.69%) and in six CWS patients (2.1% overall; two in the CWS + BGC (0.99%) and four in the CWS-BGC group (4.94%)). This difference was significant only for the CWS-BGC group versus the other two, but not significant grouped for the stent types CGuard versus CWS ± BGC (*p* = 0.49).

Asymptomatic subarachnoid hemorrhage was detected in one of the CGuard patients, in one of the CWS + BGC patients and in one of the CWS-BGC patients (the latter under triple therapy including Warfarin). One fatal parenchymal hemorrhage occurred in one of the CWS + BGC patients. The incidence of hemorrhage was not significantly different between the groups.

Follow-up ultrasound after 24 h revealed residual stenosis in six CGuard (4.2%) and in six of all CWS patients (2.1%). The differences in 24 h In-Stent stenosis grouped for the stent type were not significant (*p* = 0.49).

Most of the follow-up ultrasound examinations were performed outside of the treating hospitals. The results were only available in approximately 50% of patients. The mean delay until the last follow-up was 9.9 months for the CGuard group and 13.1 for both CWS groups. The In-Stent stenosis rate until the latest available follow-up imaging was 5.9% (four of 68 available results) for the CGuard group, 8.4% (eight of 95 available results) for the CWS + BGC group. There were no late in-stent stenoses observed in the 34 available follow-up examinations of the 81 CWS-BGC cases. Grouped for stent type there was no significant difference in the rate of stenosis in late follow-up (*p* = 0.76).

Retreatment until the latest recorded follow-up was necessary in 2.1% (3/144) for the CGuard group, 3.5% (7/203) for the CWS + BGC group and 2.5% (2/81) for the CWS-BGC group, those differences were not significant.

In the CGuard group one retreatment was performed early (within the index procedures hospital stay) due to in-stent occlusion, one retreatment was necessary due to early In-stent restenosis and one retreatment was necessary due to in-Stent restenosis detected 5 months after the initial treatment. In the CWS + BGC group no early, but seven late retreatments were necessary due to in-stent restenosis on follow-up imaging. In the CWS-BGC group both retreatments were necessary early due to early in-stent stenosis.

The mean duration of dual antiplatelet therapy differed significantly, it was 1.9 ± 1.1 months for the CGuard patients, 1.0 months without variation for all CWS + BGC patients and 2.9 ± 1.3 months for the CWS-BGC patients. However, there was no significant impact of DAPT duration on the rate of stenosis in late follow-up (*p* = 0.19) or the rate of retreatment (0.40). The rate of triple therapy including DAPT and oral anticoagulation did not differ significantly between the groups, it was given to 2.8% (4/144) patients in the CGuard group, 3.0% (6/203) patients in the CWS + BGC group and 1.2% (1/81) patients in the CWS-BGC group.

## Discussion

The best choice of treatment for carotid artery stenosis is still under debate. CAS is less invasive when compared to CEA and overall clinical results are comparable for both techniques. However, several early studies on CAS reported increased peri- and early post-procedural cerebral ischemic events, which has remained a major concern for endovascular carotid revascularization [5, 8, 13–16].

Improved stent-designs and embolic protective devices have been implemented in standard practice to address this issue. The recognition of patient selection and operator experience as influential factors as well as the implementation of quality reports have led to substantial improvements in CAS procedures overall quality [17].

Dual-layer stents are designed to improve plaque coverage and thereby decrease the risk for intra- und postprocedural thromboembolic events. The Roadsaver and CASPER-RX carotid stents were found to reduce intra-procedural cerebral emboli compared to former open-cell designs like the CWS in a randomized controlled trial [18], attributed to their reduced free cell area of 500 μm² using a nitinol mesh. Meta-analyses on available DLMS including the CGuard by Sannino et al. in 2018 as well as Stabile et al. in 2018 and 2020 showed improved clinical safety and high technical success rates for DLMS compared to the mentioned historical data on former generation carotid stents [19–21].

### Results of previous CGuard studies

In 2016 Wissgott et al. reported no procedural complications using the CGuard Stent in 30 consecutive elective patients and no minor or major stroke in their 6-month follow-up [22]. The PARADIGM study in 2016 reported 101 patients with symptomatic and asymptomatic stenoses who were treated with the CGuard; they demonstrated 99% technical success and 0% major stroke or death [23].

In the CARENET Trial CGuard was evaluated in 30 consecutive patients [24]. A 100% technical success and 0% procedural complications rate, no 30-day major adverse events were observed. On postinterventional MR Imaging after 48 h, 37% of the patients presented new ipsilateral ischemic lesions; after 30-days, all but one lesions were resolved and one new minor lesion was detected. No new ipsilateral stroke or ipsilateral stroke–related death occurred after 5 years [25].

In a series of 82 consecutive patients who were treated with the CGuard stent, technical success was 100% and one minor stroke occurred after in-stent thrombosis within 24 h [26]. MR Imaging was available in 21 of the patients and revealed new ischemic brain lesions in 23.8% of the patients. This was an improvement over the new DWI lesion rate of overall 50% (62/124) in the ICSS MRI sub-study [8] and over previous series that used older generation stent systems (e.g. PROFI trial: 45,2% with and 87,1% without proximal balloon protection [27].

The lower rates of new cerebral ischemic events are potentially attributed to the improved plaque coverage of the CGuard with a pore size of 150 to 180 μm. Several studies confirmed the benefit of plaque coverage in different novel stent designs using optical coherence tomography and demonstrated reduced plaque protrusion from 68,6% for open-cell-stents to 23,3% for closed-cell stents and 30,8% for hybrid stents [10]. For the CGuard Umemoto et al. [28] demonstrated a rate of plaque protrusion of 20,7%. However, Cappoccia et al. found no difference in terms of periprocedural emboli between CGuard and CWS in a smaller series of only 58 patients [29]. Of 733 patients who were treated with the CGuard in the IRONGUARD 2 study [30], one patient experienced fatal stroke within 30 days after the procedure and five experienced new cerebral events (four TIAs), thus resulting in a 365-day cumulative stroke rate of 0.68%. It is important to note that that the majority of the patients in this registry (92%) were treated for severe but asymptomatic carotid stenosis.

The changes induced on vascular anatomy due to stent implantation were lower for the CGuard stent when compared to the CWS and Roadsaver [11].

In the 2020 Meta-analysis on the DLMS Roadsaver and CGuard by Stabile et al. a rate of overall death and stroke of 3.77% was observed, 4.4% for Roadsaver and 3.27% for CGuard patients [21].

The CGuard was associated with a low restenosis rate of 0.65% (2 of 306 patients) within 1 year follow-up [21]. In IRONGUARD 2, the rate of restenosis for the CGuard was 0.82% (2 total occlusions and 4 in-stent restenoses) [30]. These numbers reveal an improvement over historical data from the CREST trial [3] that reported restenosis in nearly 3% of patients within 1 year, and over the rate of re-stenosis or occlusion within 2 years of 6.0% for CAS and 6.3% for CEA reported by Lal et al. in 2013 [15].

### Comparing this study with other CGuard studies

In this study the CGuard appeared safe and effective. The technical success rate in our study was excellent with 98,6% (142/144), reproducing the results of 100% technical success in the trials by Wissgot, Casana and Schofer [22, 24, 26] and 99% in the trial by Musialek [23]. Technical difficulties occurred in only three of 144 patients in the CGuard group, highly calcified high degree stenoses being the culprit in all those cases, resulting in failure of implantation in two patients and residual dissection after successful implantation and balloon PTA in another patient.

The clinical complication rate in our study was 0% in the periprocedural interval for the CGuard stent, compared with a rate of 2.9% (two TIAs and one cerebral hyperperfusion) reported on 103 CGuard patients by Tigkiropoulos et al. [31]. Abdullayev et al. compared the CGuard stent in 26 patients with the Casper-RX stent in 25 patients and the CWS in 25 patients and concluded similar safety and outcome in that rather small group of patients [32]. In the 2020 meta-analysis by Stabile et al. the combined death and stroke rate for CGuard patients during hospitalization was 1.08% (*n* = 6) and 3.27% at 1 year, with comparable results for the Roadsaver dual-layer stent [21]. Our study adds to the available data demonstrating good technical success and clinical safety results for the CGuard.

### Medical treatment

Despite the widespread execution of CAS procedures, the optimal peri- and postinterventional drug regimen after DLMS implantation remains unclear. As suggested by several guidelines, patients after CAS usually receive DAPT for at least 30 days. There is no convincing evidence on an additional benefit of DAPT over antiplatelet monotherapy later than 30 days post procedure, however there is a substantial lack of data on CAS patients with new stent designs, which is why some operators in certain situations like arterial kinking of unsatisfactory wall apposition and in presence of low individual patient risk for antiplatelet therapy might opt for prolonged DAPT. In this study there was no significant effect of the DAPT duration on the rate of In-Stent-Stenosis on latest follow-UP or the retreatment rate until latest follow-UP.

### Procedural parameters

In the IRONGUARD 2 study the authors note that omitting post-dilatation was not associated with a higher occurrence of restenosis during follow-up and concluded that post-dilatation should not be considered a mandatory part of CAS procedures with the CGuard [30].

In this study pre-dilatation was performed in about 11% of the CGuard and CWS + BGC cases but in 33% of the CWS-BGC cases due to a change in operators’ SOPs at one of the neurointerventional centers, where a routine pre-dilatation was recommended during the time of CWS-BGC interventions and the partice of unprotected predilatation was since omitted. Performing pre-dilatation did not result in an improved technical success rate; in patients with pre-dilatation, post-dilatation was performed less often (86.4%), whereas nearly all patients in the CGuard and CWS + BGC groups were treated with post-dilatation. It is our understanding that the presence of a protection with BGC or the CGuards Mesh EPS aims to reduce the overall risk of the balloon dilatation procedure and therefore increased the utilization of post-dilatation in our procedures.

The degree of carotid stenosis was significantly lower in the CGuard group (75.5% ± 14.2% for CGuards compared to 81.7% ± 11.0% for CWS + BGC and 80.3% ± 10.0% for CWS-BCG) – we attribute this to our centres positive experience with the CGuard with its mesh EPS for covering acute emboligenic lesions in thrombectomy cases with tandem occlusions leading to expanding the inclusion of patients with emboligenic lesions of intermediate stenosis degrees for elective CAS in our vascular boards.

We note higher utilization of general anesthesia for the procedures using BGC and attribute that to the fear of patient’s discomfort under balloon inflation. We observe a slightly significant negative effect of general anesthesia on the clinical complication rate (*p* = 0.045), however we hypothesize a selection bias in performing general anesthesia more often in technically challenging cases might be present.

### Restenosis

We attribute the rate of early residual stenosis of 4.2% for the CGuard and 2.1% for the CWS patients to the indication of CAS procedures including highly calcified stenoses. On 24 h follow-up duplex there was residual stenosis in six (4.2%) CGuard patients and six (2.1%) of all CWS patients. We did not systematically include information of calcification grade in our analysis, but in retrospective review of the CT-angiograms a high-grade circumferential calcification was the attributable reason for all the residual in-stent stenoses in the above cases.

The In-Stent-restenosis rate on late follow-up in our study was 6.25% (4/64) for the CGuard stent with a mean follow-up interval of 9.9 months. The rate of late In-Stent-stenosis or retreatment did not differ significantly between the CGuard and CWS groups in our study. However, the rate of available follow-up examinations was quite low (42% – 47%), this reduces the significance of the follow-up results. In their meta-analysis Stabile et al. reported a 1-year restenosis rate of 0.65% (*n* = 2) in 306 CGuard patients [21].

### Strengths and Limitations

The findings of this study should be interpreted in light of the inherent limitations of its retrospective design. Further, as in many other European studies on CAS, the proportion of symptomatic patients is quite low. This might reduce the observable benefit of using a DLMS that aims to prevent protrusion of unstable plaque, when compared with conventional carotid stents.

Clopidogrel response was not routinely assessed, so in patients experiencing adverse events or in-stent occlusions the attributable effect of Clopidogrel nonresponse remains unclear. The availability of long-term clinical follow-up data limits conclusions on long-term rates of restenosis and retreatment.

Randomized studies are needed to confirm the safety and efficacy of the CAS procedure using DLMS.

## Conclusion

In this study comparing the CGuard dual-layer mesh stent with the Carotid Wallstent we observe a favorable safety profile with a high rate of technical success, low rate of intraprocedural and early postprocedural in-stent-thrombosis, low rate of clinical complications, and low rate of restenosis on follow-up for the CGuard.

## Data Availability

No datasets were generated or analysed during the current study.
